# Insights into the RecQ helicase mechanism revealed by the structure of the helicase domain of human RECQL5

**DOI:** 10.1093/nar/gkw1362

**Published:** 2017-01-18

**Authors:** Joseph A. Newman, Hazel Aitkenhead, Pavel Savitsky, Opher Gileadi

**Affiliations:** 1 Structural Genomics Consortium, University of Oxford, ORCRB, Roosevelt Drive, Oxford OX3 7DQ, UK; 2 Structural Genomics Consortium, State University of Campinas, Campinas SP 13083-886, Brazil

## Abstract

RecQ helicases are important maintainers of genome integrity with distinct roles in almost every cellular process requiring access to DNA. RECQL5 is one of five human RecQ proteins and is particularly versatile in this regard, forming protein complexes with a diverse set of cellular partners in order to coordinate its helicase activity to various processes including replication, recombination and DNA repair. In this study, we have determined crystal structures of the core helicase domain of RECQL5 both with and without the nucleotide ADP in two distinctly different (‘Open’ and ‘Closed’) conformations. Small angle X-ray scattering studies show that the ‘Open’ form of the protein predominates in solution and we discuss implications of this with regards to the RECQL5 mechanism and conformational changes. We have measured the ATPase, helicase and DNA binding properties of various RECQL5 constructs and variants and discuss the role of these regions and residues in the various RECQL5 activities. Finally, we have performed a systematic comparison of the RECQL5 structures with other RecQ family structures and based on these comparisons we have constructed a model for the mechano-chemical cycle of the common catalytic core of these helicases.

## INTRODUCTION

RECQL5 (also known as RECQ5) is a member of the RecQ family of helicases, which are highly conserved throughout evolution (1), and function in many diverse cellular processes with the role of maintaining genome integrity. Three human RecQ genes, (BLM, WRN and RECQL4) are linked to specific disorders with symptoms of genomic instability, premature ageing and cancer disposition named Bloom's syndrome (2), Werner's syndrome (3) and Rothmund–Thomson syndromes (4) respectively. Whilst no specific human disease has been associated with mutations in RECQL5, polymorphisms have been associated with increased incidence of cancer in human populations (5,6), and knockout studies in mice show increased cancer susceptibility and greater incidence of double strand breaks and sister chromatid exchanges (7). Consistent with this it has been suggested that RECQL5 plays a general role in cells as a tumor suppressor and maintainer of genome integrity.

Specific roles have been established for RECQL5 in almost every cellular process involving DNA, including transcription, replication, recombination, chromosomal segregation and DNA repair (8). In transcription, RECQL5 is the only RecQ helicase to interact with RNA polymerase II (Pol II) (9). This interaction is direct and is formed via the kinase-inducible (KIX) domain in the C-terminus of RECQL5 (residues 515–620), which interacts with the Rpb1 Jaw domain of Pol II in a manner that resembles the transcription factor TFIIS (10). Unusually for a protein associated with Pol II, RECQL5 with was found to inhibit both the initiation and elongation of transcription (11,12), and this inhibition was not dependent on the RECQL5 helicase activity (11). The RECQL5 association, although significantly slowing the transcript elongation rate appears to reduce transcriptional stress and increase genomic stability in actively transcribed regions (12). A recent study has also established an interaction with RNA polymerase I (13), and found that the genome-stabilizing effect of RECQL5 is due to counteracting of stalled replication forks at sites of replication–transcription collisions (13). The participation of RECQL5 in other cellular processes is also largely mediated by its interactions with other protein partners, for example in replication RECQL5 has been found to interact with FEN1 (14) and PCNA (15), and is able to promote strand exchange and regression on stalled replication forks (15) and prevent replication fork defects in cells subjected to replication stresses (16,17). Furthermore, an interaction between RECQL5 and topoisomerase II α was found to stimulate its decatenation activity (18), which is required for segregation of chromosomes after replication. In recombination, RECQL5 is believed to act as an anti-recombination factor, and is able to localize to sites of laser irradiation induced single and double stranded DNA breaks (19,20). RECQL5 interacts with two important factors of early recombination, Rad51 (21,22) and the MRE11/MRN complex (23) and disrupts the Rad51 dependent formation of the nucleoprotein filament, and inhibits the exonuclease activity of MRE11, both of which are required early steps in the homologous recombination pathway.

Despite its general role as a tumor suppressor, a recent study identified a strong synthetic lethal relationship between RECQL5 and an activating V617F mutation in the JAK2 non-receptor tyrosine kinase (24), a common oncogenic lesion in patients with myeloproliferative neoplasms (MPN). RECQL5 is overexpressed in these cells and appears to contribute to maintaining their genomic stability with depletion of RECQL5 leading to increased replication stress, double strand breaks, replication fork collapse and sensitization to hydroxyurea (24) which is commonly used to treat MPN. Thus, there exists the potential to develop compounds targeting RECQL5A to treat patients with MPN.

In human cells, RECQL5 is expressed ubiquitously (25), in a cell cycle independent manner (26). Three variant forms (α, β and γ) are generated by alternate splicing. Isoforms α and γ give rise to truncated proteins (amino acids 1–410 and 1–435, respectively) that contain the N and C terminal RecA like core helicase domains, D1 and D2 (residues 1–219 and 220–364, respectively), and have strand annealing activity but no detectable helicase or ATPase activity (27). The RECQL5 β isoform, which includes a conserved Zn^2+^ binding subdomain (resides 365–437) that was found to be essential for efficient helicase activity (27), and an extended C-terminus (residues 438–991), is the active form of the protein and the focus of this study, and will be referred to hereinafter as simply RECQL5. The C-terminal region of RECQL5 does not contain significant homology to other RecQ family helicases, and appears to contain the regions responsible for the binding of various cellular partners. These include the KIX domain and the Set2-Rpb 1-interacting (SRI) domains (residues 515–620 and 900–991, respectively) that enable RECQL5 to interact with Pol II (10,28), the region responsible for RAD 51 interaction (21,22) (residues 654–725), a putative PCNA interacting region (residues 964–971) (15), and a nuclear localization signal located within in the C-terminal 240 amino acids (29).

In this study, we present two structures of the RECQL5 catalytic core, obtained in two distinctly different conformational states that have been crystallized in the presence and absence of ADP/Mg^2+^ to a resolution of 1.8 and 3.4 Å, respectively. Analysis of the structures and comparisons with other RecQ family members provides insights into the role of RECQL5 in the maintenance of genome integrity and the conformational changes associated with nucleotide binding and hydrolysis. Solution studies show that an open form of the RECQL5 structure is predominant in solution, and the conformational switching between open and closed forms may be a distinct feature of the RECQL5 mechanism. We demonstrate through enzymatic assays that a RECQL5 specific α-helical extension to the Zn^2+^ binding domain is essential for helicase activity and have measured the activities of various mutants identified in our structures to contribute towards energy coupling and discuss these activities with respect to a possible RECQL5 helicase mechanism. Finally we have performed a quantitative structural classification and comparison of all current RecQ family member structures, and discuss similarities and differences of the various conformational states and their implications for the general RecQ family helicase mechanism.

## MATERIALS AND METHODS

### Cloning overexpression and purification

RECQL5 constructs were cloned in to the pNIC28-Bsa4 vector for histidine tagged overexpression in *Escherichia coli*. The RECQL5 11–526 variants were generated from the WT construct by a site directed mutagenesis strategy. Proteins were expressed in BL21(DE3)-R3-pRARE following IPTG induction at 18°C. For purification, cell pellets were thawed and resuspended in buffer A (50 mM HEPES pH 7.5, 500 mM NaCl, 5% glycerol, 10 mM imidazole, 0.5 mM Tris (2-carboxyethyl) phosphene (TCEP)). Cells were lysed using sonication and cell debris pelleted by centrifugation. Lysates were applied to a Ni-IDA IMAC gravity flow column, washed with two column volumes of wash buffer (buffer A supplemented with 45 mM imidazole), and eluted with the addition of 300 mM imidazole in buffer A. The purification tag was cleaved with the addition of 1:20 mass ratio of His-tagged TEV protease during overnight dialysis into buffer B (20 mM HEPES, pH 7.5, 500 mM NaCl, 5% glycerol, 0.5 mM TCEP). TEV was removed by IMAC column rebinding and final protein purification was performed by size exclusion chromatography using a HiLoad 16/60 Superdex 200 column at 1 ml/min in buffer B. Protein concentrations were determined by measurement at 280 nm (Nanodrop) using the calculated molecular mass and extinction coefficients, and intact masses were confirmed by ESI-MS. Coomassie stained gels of all constructs used in this study are shown in Supplementary Figure S1.

### Crystallization and structure determination

For crystallization of the APO form (construct RECQL5 11–526) proteins were concentrated to 20 mg/ml using a 50 000 MWCO centrifugal concentrator and crystals were obtained by hanging drop vapor diffusion in conditions containing 17.5% PEG 3350, 0.2 M potassium thiocyanate, 0.1 M HEPES pH 7.0, 10% ethylene glycol. ADP and MgCl_2_ were added at a final concentration of 5 mM and the monoclinic form (construct RECQL5 11–453) was crystallized at 15 mg/ml by sitting drop vapor diffusion from conditions containing 19% PEG 6K, 0.25 M lithium chloride and 10% ethylene glycol. Crystals of the triclinic form were obtained in conditions containing 0.1 M Tris pH 7.5, 23% PEG 3350 and 0.1 M NaCl. The APO and monoclinic ADP form crystals were cryo-protected by transferring to a solution of mother liquor supplemented with 20% ethylene glycol, whilst the triclinic ADP form was cryo-protected in a solution containing well solution supplemented with 10% DMSO and 10% ethylene glycol. Data were collected at Diamond Light Source beamlines I04 (APO form), I04-1 (triclinic ADP form) and I03 (monoclinic ADP form). Diffraction data were processed with the program XDS (30), and the structures were solved by molecular replacement using the program PHASER (31) with the BLM helicase (32,33) structure as a starting model. Model building and real space refinement were performed in COOT (34) and the structures refined using BUSTER (APO and triclinic ADP form) and PHENIX REFINE (35) (monoclinic ADP form).

### Small angle X-ray scattering

Small angle X-ray scattering measurements of RECQL5 in solution were performed at Diamond Light Source beamline B21 using a BIOSAXS robot for sample loading. Measurements were made using protein concentrations of 4, 2 and 1 mg/ml in a buffer comprising 20 mM HEPES pH 7.5, 250 mM NaCl, 1 mM TCEP. Nucleotides were added to a final concentration of 1 mM, and DNA substrates (single stranded 19 mer) were added in a 1:1.2 molar ratio of Protein:DNA. The data were reduced and buffer contributions subtracted with the DawnDiamond software suite and analysed using the program SCATTER (www.bioisis.net). Scattering profiles of atomic models were calculated from atomic models using CRYSOL (36) and the agreement between theoretical and experimental scattering profiles was evaluated using the χ2 free procedure (37) implemented in the program SCATTER. Distance distribution functions were calculated using SCATTER and used for subsequent *ab inito* shape reconstructions using the program DAMMIF (38). The results of 13 parallel reconstructions were compared using the program SUPCOMB (39) and the model with the lowest normalized spatial discrepancy was chosen for analysis. Atomic models were fit to the SAXS envelopes using the program SUPCOMB.

### DNA binding assays

DNA binding was measured using a fluorescence polarization based assay. DNA oligonucleotide substrates were prepared by mixing the oligonucleotide sequences and combinations listed in Supplementary Table S1. For all substrates, the master strand was labelled on the 5΄ end with flourescein isothiocyanate and oligonucleotides were mixed in the ratio 1:1.1 (slight excess of unlabeled oligonucleotide) at 10 μM final concentration in a buffer consisting of 10 mM HEPES pH 7.5, 50 mM NaCl before heating to 96°C and allowing to cool on a heat block over 2 h. Probes were used at a final concentration of 10 nM and binding experiments were performed in a buffer containing 10 mM HEPES pH 7.5, 150 mM NaCl. Measurements were performed in 384-well plates (30 μl volume) at 25°C in a POLARstar plate reader (BMG Labtech). Kinetic constants were calculated from binding curves using a four-parameter logarithmic binding equation using the program PRISM (GraphPad).

### ATPase activity assays

ATPase activity was measured using a pyruvate kinase, lactate dehydrogenase enzyme linked absorbance assay (40) in which the loss of absorbance at 340 nm (6250 cm^−1^ M^−1^) is coupled to ATP hydrolysis. The reaction mixtures (40 μl) contained 0.85 U lactate dehydrogenase, 4.2 U pyruvate kinase, 0.2 mM NADH, 0.5 mM phosphoenolpyruvate, 20 mM HEPES pH 7.5, 200 mM NaCl and ATP concentrations between 3 μM and 0.8 mM. For DNA stimulated ATPase, the reaction mix contained an additional 2 μM single stranded 61 nt DNA (5΄-GACGCTGCCGAATTCTACCAGTGCCTTGCTAGGACATCTTTGCCCACCTGCAGGTTCACCC). Reactions were initiated by the addition of protein (20 nM with DNA and 1 μM without) and initial rates were measured at 37°C in a POLARstar omega plate reader (BMG Labtech). Kinetic constants were calculated from binding curves using a four-parameter logarithmic binding equation using the program PRISM (GraphPad).

### Helicase assays

One strand of the splayed duplex DNA substrate (RQ1 5΄ATCGATAGTCGGATCCTCTAGACAGCTCCATGTAGCAAGGCACTGGTAGAATTCGGCAGCGTC) was end labelled using polynucleotide kinase and γ-^32^P-labeled ATP. A 2-fold excess of unlabeled complementary DNA was added (RQ3 5΄GACGCTGCCGAATTCTACCAGTGCCTTGCTAGGACATCTTTGCCCACCTGCAGGTTCACCC) and substrates were heated to 95°C and allowed to cool slowly to room temperature. Probes were purified by applying to a micro bio spin 6 column (Bio-Rad). Probes were added at a final concentration of 0.1 nM in a 10 μl reaction mixture containing 20 mM HEPES pH 7, 5 mM ATP, 10 mM MgCl_2_, 1 mM DTT, 10% glycerol, 50 mM KCl, 10 nM unlabeled RQ1 DNA (added to prevent re-annealing of labelled probe) and varying concentrations of RECQL5 protein from 5 μM to 40 nM. Reactions were incubated for 60 min at 25°C and stopped by the addition of 10 μl stop buffer (0.9% SDS, 25 mM EDTA, 10% glycerol and 0.05% bromophenol blue). Reaction products were separated with 12% acrylamide gel in TBE buffer at 170 V for 70 min. Gels were dried and exposed overnight on a phosphor-imaging screen. Quantification was performed using Bio-Rad imagelab software.

### Strand annealing assays

^32^P-labeled RQ1 substrate was prepared as for the helicase assay. Reaction mixtures (10 μl final volume) contained 100 nM RECQL5 protein, 0.8 nM Radiolabelled RQ1 probe, 0.8 nM unlabelled RQ3 (splayed duplex) or RQ2 probe (double stranded), in a buffer containing 20 mM HEPES pH 7, 5 mM ATP, 10 mM MgCl, 1 mM DTT, 10% glycerol and 50 mM KCl. Reactions were incubated at 37°C and 10 μl stop buffer was added at time points of 2, 5, 10, 20, 40 and 60 min. Reaction products were separated and imaged as for the helicase assays.

## RESULTS AND DISCUSSION

### Quality of the model

**Figure 1.**
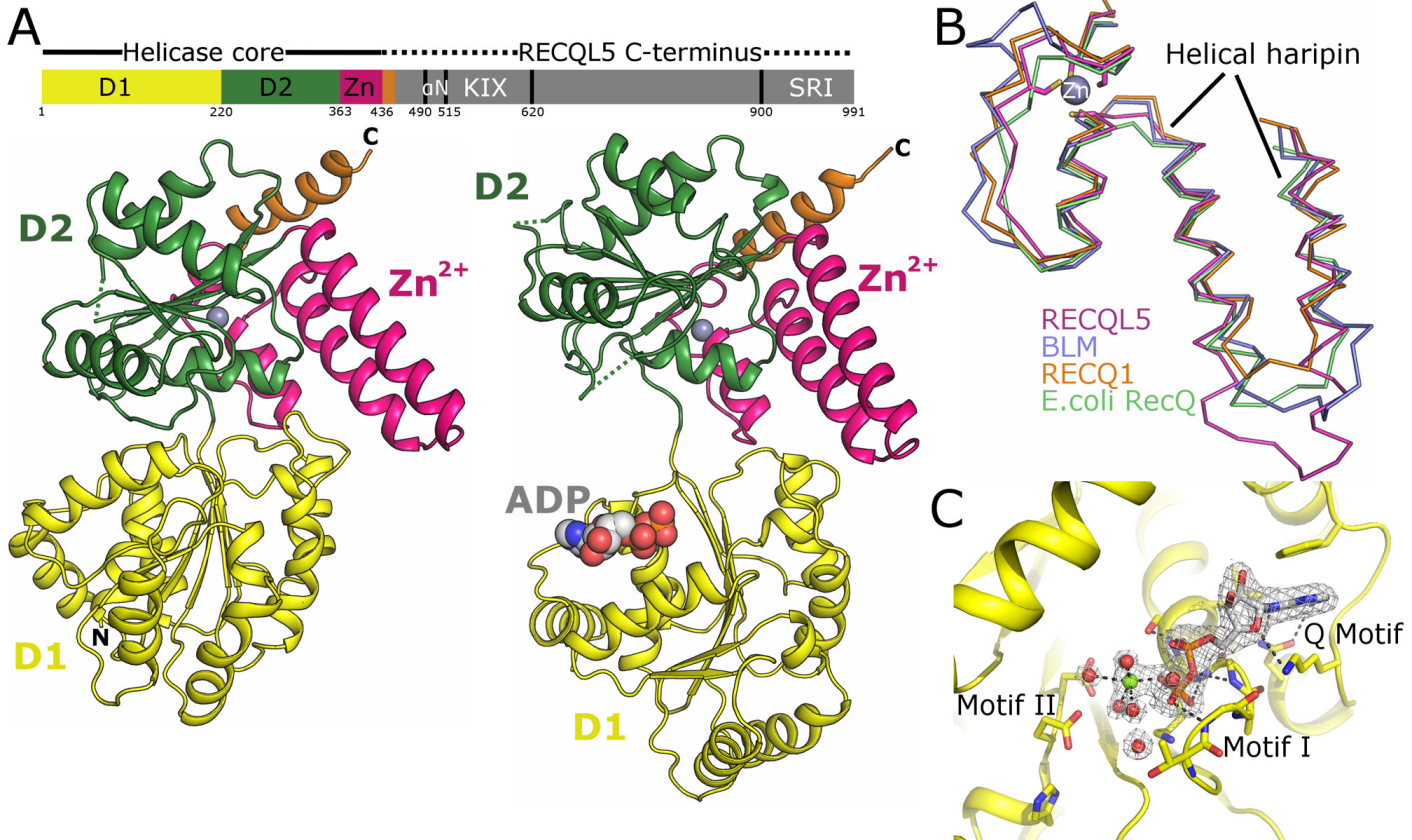
Structure of APO and ADP bound RECQL5. (**A**) Cartoon diagram of the APO (left) and ADP bound (right) RECQL5 forms with secondary structural elements colored by domain and the Zn^2+^ ion shown as a grey sphere. The top panel shows a schematic view of the domain arrangement in full length RECQL5. (**B**) Comparison of the Zn^2+^ binding subdomain across the RecQ family, the helical hairpin in RECQL5 is significantly longer than in other family members. (**C**) Close up view of the nucleotide binding site in the ADP form crystals, the ADP is shown is stick representation with polar contacts shown as black dashed lines and the 2*F*_o_ – 1*F*_c_ electron density map (contoured at 1σ) shown in grey covering the ADP/Mg^2+^.

Crystals of the Apo form of RECQL5 were obtained using a construct spanning residues 11–526, this includes both D1 and D2 RecA like domains, the Zn^2+^ binding subdomain, and a short section (residues 438–453) of the previously uncharacterized RECQL5 C-terminal region (Figure 1A). Crystallization experiments were performed by sitting drop vapor diffusion with RECQL5 both alone and in complex with the nucleotides ADP, ATP-γS and AMP-PCP. Crystals were obtained for the APO protein which diffracted to a maximum resolution of 3.2 Å, and the structure was solved by molecular replacement using the structure of BLM helicase as a search model (32), with two chains in the asymmetric unit. The electron density map is of overall reasonable quality although the relatively low resolution (3.4 Å) and high Wilson B factor ∼110 Å^2^) make the map difficult to interpret in some areas. A sharpening *B* factor of around 80 Å^2^ was found to aid map interpretation, revealing side chain electron density in many areas, although even after this correction a number of sidechains were truncated in the worst areas. The model is complete from residues 12–452, with the exception of loops 250–260 and 321–323 which could not be modeled due to disorder. The final 74 residues at the C-terminus are absent from the model, and appear to be completely disordered from the electron density maps, indicating this region is disordered, consistent with the results of various disorder prediction servers.

In an attempt to improve the diffraction properties of these crystals a new construct spanning residues 11–453 was produced and crystallization experiments were set up with and without nucleotides as above. In this case crystals were obtained in complex with the nucleotide ADP/Mg^2+^ in two different crystal forms, both of which diffract to significantly higher resolution (1.8 Å for the monoclinic crystals, and 2.0 Å for the triclinic). The ADP bound structures were solved by molecular replacement using the APO form as a search model, with two RECQL5 monomers in the asymmetric unit of the monoclinic crystals, and four copies in the triclinic crystals. In both cases, the electron density maps are of excellent quality throughout and the structures are very similar, although slight differences remain in the domain orientation and conformation of various loops which are discussed in more detail below. All models have been restrained to standard bond lengths and angles with good geometry statistics (Table 1).

**Table 1.** Data collection and refinement statistics

	APO form	ADP/Mg^2+^ monoclinic	ADP/Mg^2+^ triclinic
Space group	*P*4_3_2_1_2	*P*2_1_	*P*1
Cell dimensions, *a, b, c* (Å)	68.7, 68.7, 396.3	72.8, 62.9, 104.7	63.6, 84.8, 106.7
Angles α, β, γ (°)	90, 90, 90	90, 95.4, 90	108.8, 90.1, 96.9
Wavelength (Å)	0.979	0.976	0.928
Resolution (Å)	48.6–3.40 (3.67–3.40)	19.8–1.80 (1.83–1.80)	100.9–2.00 (2.03–2.00)
*R*_merge_	0.120 (1.183)	0.098 (1.01)	0.114 (1.510)
*R*_p.i.m._	0.045 (0.44)	0.058 (0.60)	0.059 (0.766)
*I*/σ*I*	13.7 (2.1)	8.8 (1.3)	12.4 (1.5)
CC1/2	0.995 (0.895)	0.996 (0.658)	0.993 (0.402)
Completeness (%)	99.9 (99.8)	99.8 (99.9)	99.0 (99.0)
Multiplicity	8.2 (7.5)	3.8 (3.8)	4.6 (4.7)
No. of unique reflections	14 135 (2798)	87 336 (4418)	139 875 (6942)
Refinement statistics			
Resolution	48.6–3.4	19.88–1.80	100.8–2.00
*R*_work_/*R*_free_ (%)	30.14/32.18	19.49/23.44	24.4/28.2
No. of atoms			
Protein	6564	6883	13 859
Solvent	—	1000	399
Ligand/ion	—	58	128
Average *B* factors (Å^2^)			
All atoms	145	37	56
Protein	145	36	57
Solvent	—	41	45
Ligand/ion	—	37	59
Wilson *B*	98	25	38.5
R.M.S. deviations			
Bond lengths (Å)	0.008	0.004	0.010
Bond angles (°)	1.0	0.79	1.05
Ramachandran plot			
Favored (%)	94	98	98
Allowed (%)	100	100	100
PDBid	5LB8	5LB3	5LB5

Values in parenthesis refer to the statistics in the highest resolution shell.

### Structure of the RECQL5 catalytic core

Both Apo and ADP bound crystal forms have the same basic architecture, with the two RecA-like domains D1 and D2 each consisting of a central 6- or 7-stranded parallel beta sheet flanked on either side by α-helices (Figure 1A). The Zn^2+^ binding subdomain follows immediately after, and is closely associated with the D2 domain. The Zn^2+^-binding domain consists of a pair of long antiparallel α-helices (also known as the helical hairpin), followed by series of short helices and loops, from which a cluster of cysteine residues bind in a typical Cys4 tetrahedral arrangement to a single Zn^2+^ ion (Figure 1B). When examined in isolation the individual D1 and D2 domains in RECQL5 are very similar both to each other (generally around 1.0 Å RMSD) and to the equivalent domains in other RecQ family helicases (generally around 1.5 Å RMSD), with the exception of a seven residue insertion between the first α-helix and second β-strand of the D2 domain which is longer in RECQL5 and occupies a position close to where the HRDC domain of BLM protein (which does not occur in RECQL5) packs against the D1 and D2 domains (32) (Figure 1A). The Zn^2+^ binding subdomain is more varied across the family; the helical hairpin motif of RECQL5 contains an additional turn of α-helix, making it significantly longer than other RecQ family structures solved to date (Figure 1B).

In the ADP complex, the ADP moiety is bound in the active site, making extensive interactions with residues in the D1 domain, several of which belong to the conserved helicase motifs Q, I and II (Figure 1C). More specifically the adenine base is sandwiched between the side chains of Phe 26 and Lys 30, making π–π stacking interactions with the former and van der Waals interactions with the aliphatic region of the latter. Gln 34 (the Q of the ‘Q motif’, also known as helicase motif 0), accepts and donates hydrogen bonds to the N6 and N7 nitrogen's respectively, conferring nucleotide specificity to the binding site. The ribose makes a single hydrogen bond via O4΄ with Lys 30 and an additional possible water mediated contact with His 89. The α and β phosphates are bound in the classic Walker A or P-loop configuration (also known as helicase motif I), making hydrogen bonds to five successive main chain nitrogens, bounded on the far side by Lys 58. The binding site is completed by a number of bound waters and a hydrated Mg^2+^ ion, which is well defined in the electron density and makes a number of water mediated interactions with D157 and E158, and is suitably positioned to interact with oxygens from both the β and γ phosphates of ATP (Figure 1C).

### Comparisons of APO and ADP bound forms

**Figure 2.**
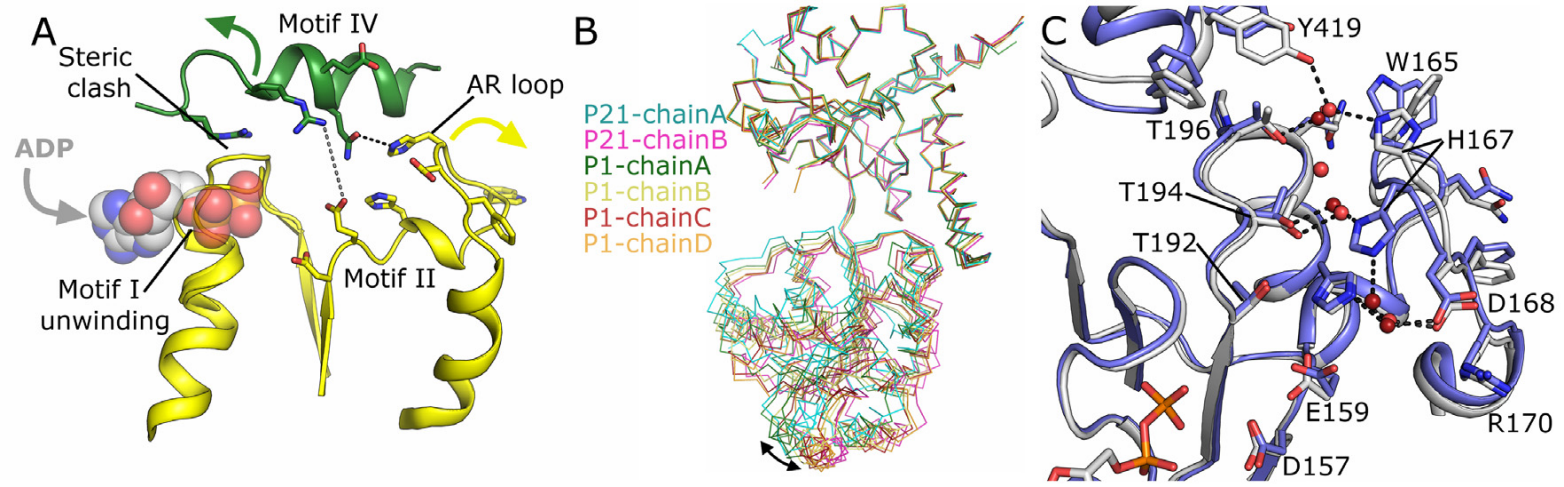
Comparisons of various RECQL5 crystal forms. (**A**) Close up view of the active site showing a possible structural transition of motifs I, II, IV and the AR loop following nucleotide binding. (**B**) Comparison of the conformation of the six various chains of the monoclinic and triclinic ADP form crystals. (**C**) Close up view of the AR loop which was found to occupy two distinct states in the ADP form crystals, with H167 forming water mediated interactions with either Y419 (part of the Zn^2+^ domain, gray carbons) or T194 (part of motif III, blue carbons).

Comparing the various ADP and APO crystal forms has allowed us to identify both local and global changes that may be relevant to the RECQL5 catalytic cycle. The most prominent difference is in the relative orientations of the D1 and D2 domains, with the inter-domain cleft almost completely closed in the APO form and considerably more open in the ADP bound form, with maximal displacements of equivalent residues of up to 30 Å (Figure 1A). On a local level, the residues forming the bulk of the nucleotide contacts from motif I undergo a significant helix to loop transition, exchanging several hydrogen bonds from canonical α helical hydrogen bonds, to phosphate oxygen contacts, with the entirety of residues 53–55 shifted significantly to accommodate the nucleotide (Figure 2A). This type of structural rearrangement is very similar to that seen when comparing the APO and nucleotide bound forms of RecQ from *Deinococcus radiodurans* (41), and *E. coli* (42). Following nucleotide binding a significant movement of D2 would be required either to alleviate steric clashes with the shifted residues in the ADP bound conformation (as seen when mapping this transition on to APO RECQL5 (Figure 2A)), and/or to form polar interactions with the phosphates (as seen in the crystal structure of BLM in complex with ADP (32)).

Similar but less dramatic differences can be seen when comparing the six independent chains of the two ADP crystal forms, which can be grouped into two distinct clusters of three which are separated by maximum displacements of up to 9 Å (Figure 2B). Looking at residues at the interface between the two domains, a clear correlated difference can be seen in the conformation of residues forming the aromatic rich loop (AR loop). In the more open form H167 and D168 are pointing toward motif II, making water-mediated interactions with T194 and H160 (conserved residues from motifs III and II respectively), while in the less open form H167 is pointing away from motif II, forming a water-mediated interaction with Y419 (part of the Zn^2+^ binding domain) (Figure 2C).

### Small angle X-ray scattering (SAXS) studies of RECQL5 in solution

**Figure 3.**
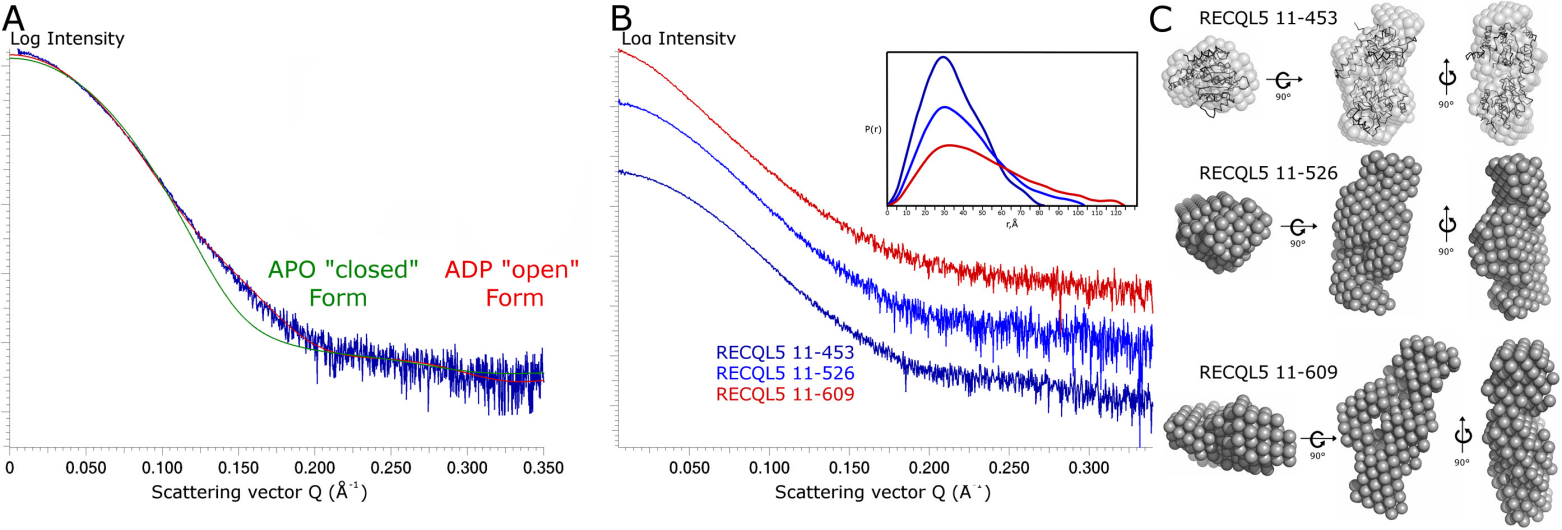
SAXS analysis of RECQL5 in solution. (**A**) Comparison of the theoretical (APO form in green and ADP form in red) and experimental scattering curves for the RECQL5A 11–453 construct, the experimental data shown was collected in the absence of nucleotide at 2 mg/ml. (**B**) SAXS scattering curves of the various length RECQL5 constructs follow a broadly similar profile. *P*(*r*) distance distribution functions (shown in the inset), and *ab initio* shape reconstructions shown in panel (**C**), are consistent with an increasingly elongated particle with similar cross sectional area.

In order to assess the conformational state of RECQL5 in solution, we have performed SAXS analysis on a number of RECQL5 truncated constructs both in the presence and absence of various nucleotides and single stranded DNA. Comparing the scattering curves in the presence and absence of the nucleotides ADP or the ATP analogue AMP–PNP reveals only very minor differences (a quantitative comparison with the program Vr (43) reveals that all curves have Vr similarity scores of around 1.0, and Rg values within 2.0 Å (Supplementary Figure S2)). This is in contrast to the situation for the BLM helicase, for which significant differences could be observed upon nucleotide binding (32), which were attributed to movements in the HRDC domain not present in RECQL5. Comparing the scattering curves of the 11–453 construct with calculated scattering curves from the atomic models of RECQL5 reveals an excellent agreement with the open form monomer (Chi^2^ = 0.33 Chi^2^free = 0.46), whilst the agreement with the closed conformation is significantly worse (Chi^2^ = 1.82 Chi^2^free = 2.35), (Figure 3A), indicating that the open conformation is the predominant form in solution in both the presence and absence of nucleotide.

### The RECQL5 C-terminal region

We have also used SAXS analysis to probe the structural nature of the RECQL5 C-terminus, which in RECQL5 extends for a further ∼550 residues from the Zn^2+^ domain, and in contrast to most other human RecQ family helicases lacks a winged helix (WH) or HRDC domain. The majority of the C-terminal region is poorly characterized, although two regions of the RECQL5 C-terminus have been characterized biochemically to be important for mediating the interaction with RNA polymerase II (10), the KIX domain (residues 515–620) and the SRI domain (residues 900–991). We were specifically interested in the question of whether RECQL5 possesses an analogous structure to the WH domain which has been shown in other RecQ family members to bind to the double stranded DNA region and provide an equivalent to the β-hairpin ‘wedge’ to aid in DNA duplex separation.

To further probe this region additional SAXS data were collected on RECQL5 constructs corresponding to, 11–453, 11–526 and 11–609. All three constructs gave broadly similar scattering profiles, with only minor changes associated with the addition of nucleotides and Rg and Vc values increasing in accordance with the molecular weight (Figure 3B). A*b-initio* shape reconstructions indicate that the constructs share a common overall shape, which for the 11–453 construct is a good agreement with the crystal structure (Figure 3C). The longer constructs show increasingly elongated shape with addition of residues from the C-terminal region, this is consistent with the residues in region 453–609 being generally globular and extending from the current C-terminus without looping back and associating with the helicase core as is the case for the HRDC domain in other RecQ helicases (Figure 3B).

### The RECQL5 C-terminal ‘wedge’ helix is essential for helicase activity

**Figure 4.**
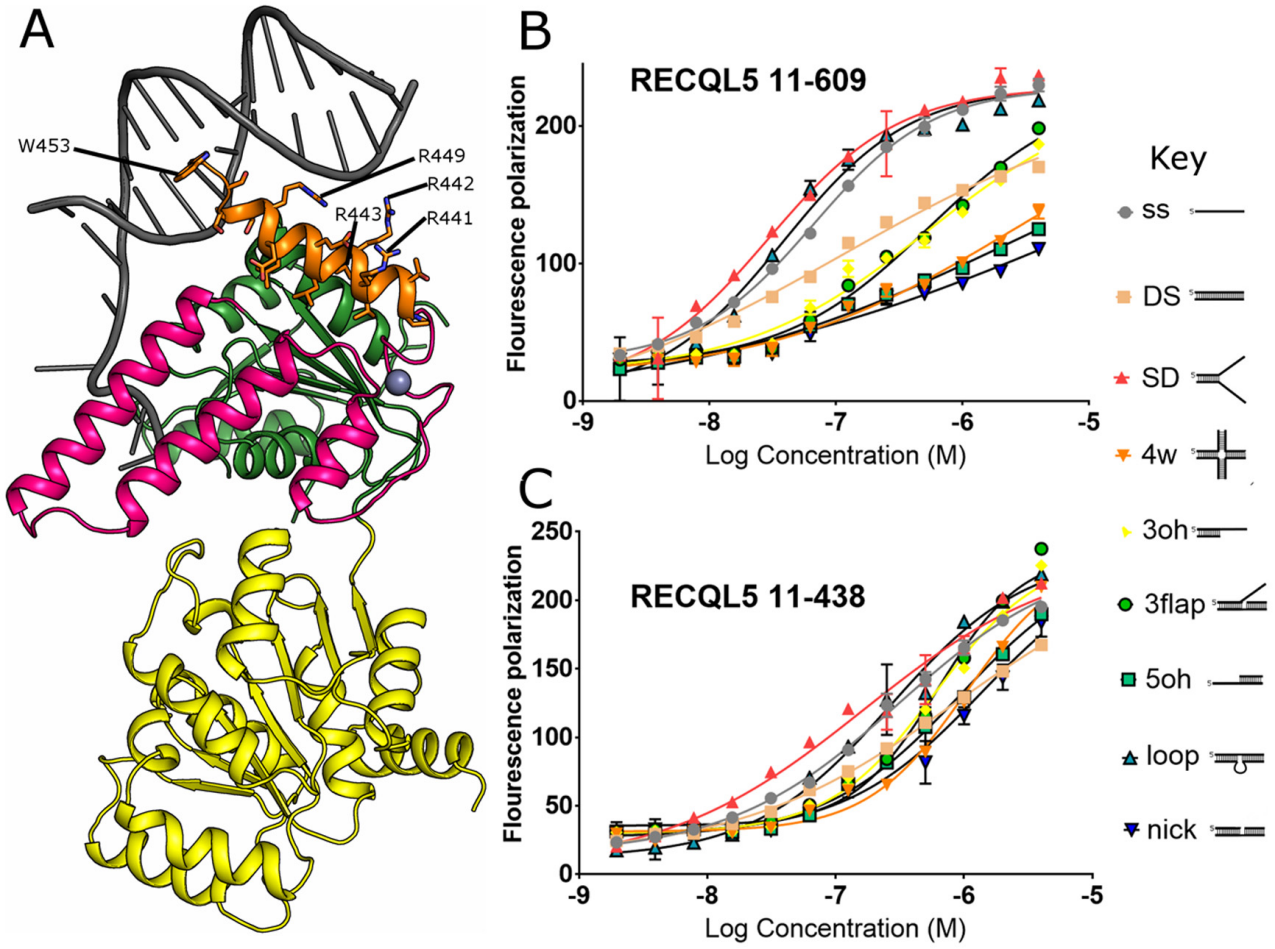
Role of the RECQL5 ‘wedge’ helix in DNA binding. (**A**) Model of possible RECQL5 DNA complex based on a structural superposition with the BLM DNA complex, residues in the C-terminal ‘wedge’ helix that may contribute to DNA binding are labelled and shown in the stick format. (**B**) Characterization of RECQL5 11–609 DNA binding affinity and specificity by fluorescence polarization, showing a clear preference for binding splayed duplex, looped or single stranded DNA. (**C**) The RECQL5 11–438 construct lacking the ‘wedge’ helix had significantly reduced affinity and specificity. For all graphs, error bars are plotted as ±S.E. from three replicates. In the case of the 11–438 data, curves for all substrates were fitted with a single shared *B*_max_ to ensure a more robust fit.

In both the APO and ADP bound RECQL5 structure a single additional α-helix can be seen following on from the Zn^2+^ binding domain, which points roughly orthogonal to the helical hairpin and occupies a broadly similar position to the WH domain found in the C-terminal regions of other RecQ helicases (44). Interestingly, this helix contains a cluster of positively charged residues (R441, R442, R443 and R449), indicating the potential to form polar contacts to the DNA backbone, and has an aromatic residue W453 in a solvent exposed position roughly equivalent to the position of the β-hairpin moiety in other RecQ helicases (Figure 4A).

To further probe the function of this helix we have measured the DNA binding, ATPase and helicase activities of constructs with and without this feature. The DNA binding activity was measured by fluorescence polarization assays against a variety of DNA substrates, (Figure 4B, Supplementary Figure S2). The 11–609 construct showed tight binding of around 20–50 nM to splayed duplex and looped duplex substrates, slightly weaker binding (approximately 2–4 fold) to single stranded and double stranded DNA, whilst the remaining substrates gave apparent dissociation constants around the low micro-molar range (Figure 4B). The fact that RECQL5 shows significantly higher affinity for splayed duplex substrates than either 5΄ or 3΄ overhangs is significant, and may indicate an additional means by which RECQL5 could be targeted towards either replication or transcription. This general pattern of selectivity is conserved across the shorter constructs with similar apparent binding affinities for the most favoured substrates (Supplementary Figure S3). The apparent binding affinity of RECQL5 11–438 (which contains a complete Zn^2+^ binding subdomain but lacks the putative DNA binding helix) was however significantly lower than all other constructs (kD app > 150 nM), and did not show as pronounced selectivity (Figure 4C and Table 2), indicating that the helix immediately C-terminal to the Zn^2+^ binding domain plays an important accessory role in DNA binding.

**Table 2.** Kinetic properties of the various RECQL5 constructs and variants

Construct/variant													
Activity	11–609	11–526	11–453	11–438	11–526 (W165A)	11–526 (H167A)	11–526 (D168A)	11–526 (Q345A)	11–526 (R349A)	11–526 (R352G)	11–526 (Y419A)	11–526 (F420A)	11–526 (D422A)
DNA binding (SD) *K*_D_ (nM)	29 ± 4	51 ± 5	49 ± 28	164 ± 34	169 ± 6	108 ± 5	25 ± 2	85 ± 4	295 ± 29*	29 ± 2	39 ± 10	45 ± 3	38 ± 4
ATPase (min^−1^)	ND	0.72 ± 0.06	ND	ND	0.56 ± 0.09	0.64 ± 0.07	2.98 ± 0.21	1.53 ± 0.08	—	0.27 ± 0.09	1.36 ± 0.08	—	0.22 ± 0.06
DNA stimulated ATPase (min ^−1^)	631 ± 24	1146 ± 60	600 ± 50	877 ± 48	836 ± 46	702 ± 58	856 ± 56	90 ± 22	24 ± 8	57 ± 36	648 ± 27	480 ± 19	670 ± 46
DNA stimulated ATP *K*_m_ (μM)	43 ± 6	87 ± 15	175 ± 40	87 ± 15	170 ± 31	100 ± 25	20 ± 6	225 ± 157	312 ± 247	751 ± 869	130 ± 17	165 ± 19	192 ± 40
Helicase activity	+++	++	++	−	−	−	++	−	−	−	++	+	+
Annealing	+++	+++	+++	+++	+++	+++	+++	+++	+++	+++	+++	+++	+++

ND = not determined, +++ = highest activity, ++ = Slight reduction in activity, + = significant reduction in activity, – = no measurable activity.

*The R349A mutant was found to have an additional substitution R267W introduced by PCR in the cloning process. It is believed this may affect the DNA binding but not the ATPase activities.

**Figure 5.**
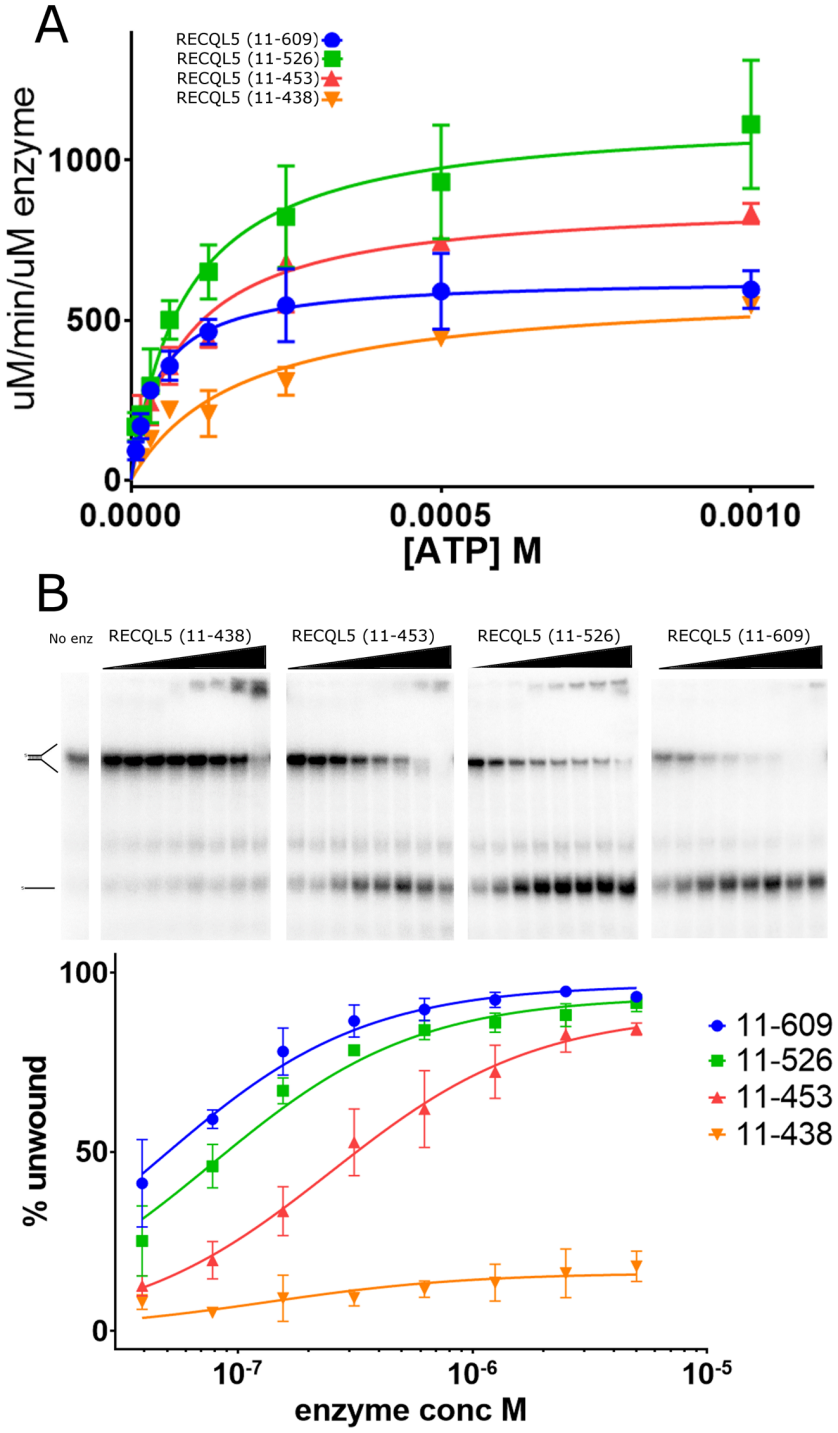
Characterization of the ATPase and Helicase activities of the various RECQL5 constructs. (**A**) DNA stimulated ATPase activity of RECQL5 constructs plotted as a function of ATP concentration. (**B**) Helicase activity of RECQL5 constructs, the upper panel shows representative gels and the lower panel shows quantification based on three independent experiments with error bars are plotted as ±S.E.

The ATPase activity of constructs 11–438, 11–453, 11–526 and 11–609 were roughly similar with Km values in the range of 50–150 uM and Kcat values varying by a maximum of around 2 fold between 10 sec-1 and 20 sec-1 (values represent activity with stimulation by 2μM single stranded DNA) (Figure 5A). The helicase activity of these constructs was measured using a splayed duplex DNA substrate over a range of protein concentrations to help distinguish helicase and DNA binding activities (Figure 5B). In general agreement with the DNA binding results the 11–609 construct had robust helicase activity in our assay when used at concentrations over 50 nM, with the 11–499 and 1–453 constructs both showing a slight reduction in activity relative to the longer construct. The 11–438 construct however displayed an almost total lack of helicase activity with no significant activity detected up to a concentration of 5 μM (Figure 5B). This effect is much greater than the reduction in either DNA binding or DNA stimulated ATPase activity, indicating that the putative DNA binding helix plays a crucial role in helicase activity. To gain further insights into the nature of the interactions with DNA we created a two further constructs which were truncated at residues 446 (lacking the C-terminal half of this helix) and 452 (lacking the putative base pair interacting aromatic W453) and assayed their helicase activity (Supplementary Figure S4). Both constructs show some activity compared to the inactive 11–438, although the activity is significantly reduced (<20%) compared to the parent 11–453 construct. Thus, it appears that residues in the C-terminal of this helix in particular W453, while not being absolutely essential for activity play a significant role in helicase activity. Comparisons with other helicases obtained in complex with DNA show that this region is likely to be in close contact with the double stranded portion of the DNA substrate (Figure 4A), possibly acting as a wedge to aid in duplex separation, therefore we have named this helix the ‘wedge’ helix. Alternatively, this helix may aid strand separation by defining a greater ‘break angle’ between the double and single stranded DNA paths in a similar mechanism to that proposed for bacterial RecQ (45).

### Analysis of possible energy coupling mutations

**Figure 6.**
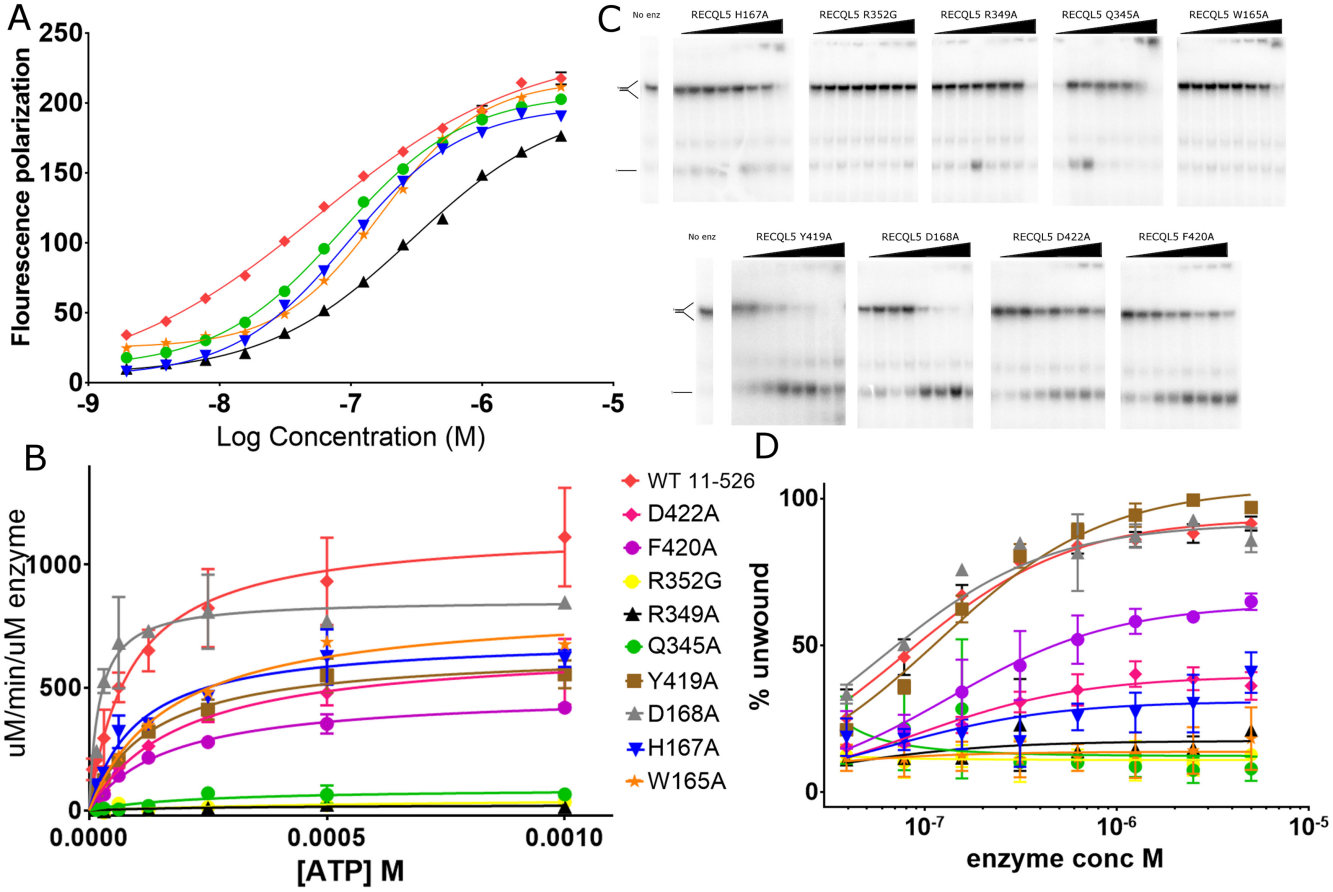
Characterization of the DNA binding, ATPase and helicase activities of RECQL5 variants. (**A**) DNA binding activity of RECQL5 variants binding to a splayed duplex DNA probe, only variants with significant difference from WT are shown for clarity. (**B**) DNA stimulated ATPase activity of RECQL5 variants plotted as a function of ATP concentration. (**C**) Helicase activity of RECQL5 variants, the upper panel shows representative gels and the lower panel shows quantification based on three independent experiments. The symbol scheme is shown on the bottom left hand side of panel and was used for all three plots and graphs are plotted as mean ±S.E. from three replicate experiments.

We have constructed a number of point mutants of RECQL5 that feature substitutions in residues that would appear from our structures to be important for conformational changes and are located at the D1–D2 interface in either the open or closed structures. We have chosen three residues each from motif VI, the AR loop, and the Zn^2+^ binding region (Supplementary Figure S5), and have tested the DNA binding, ATPase and Helicase and strand annealing activities of these mutants (Figure 6 and Table 2). The DNA binding activity for the mutants was mostly similar to WT (Figure 6A, Supplementary Figure S6), with the exception of three mutants (H167A, W165A and Q345), two of which are located in the AR loop and displayed a moderate reduction in affinity (2–3-fold), consistent with these residues contributing towards DNA binding as was found to be the case for the *D. radiodurans* RecQ DNA complex structure (41). The ATPase activity was measured both in the presence and absence of single stranded DNA, to test for possible coupling mutations. In the absence of DNA the Km for ATP appears to be largely unchanged at around 50 μM (value was fit globally to all data to give more robust fit), with *K*_cat_ values around 1 min^−1^ (∼600-fold reduction over DNA stimulated ATPase) for the WT construct (Figure 6B, Supplementary Figure S7).

Seven of the nine mutants had either a complete loss or a significant reduction in helicase activity, with only D168A and Y419A displaying helicase activity comparable to WT (Figure 6C, D and Table 2). The three mutants from motif VI with no helicase activity can be explained by a significant reductions in DNA stimulated ATPase activity R349A, R349G and Q345A (Figure 6C), with the latter mutant appearing to be deficient in the capacity for stimulation of ATPase activity by DNA, due to the fact is not deficient at ATP hydrolysis in the absence of DNA. Two residues at the AR loop region, W165 and H167 are also essential for helicase activity, although interestingly these variants have DNA stimulated ATPase activity similar to WT. Given the fact that the DNA binding affinity of these variants is somewhat reduced, it is likely that they play a role in grasping the DNA in a manner suitable for productive helicase activity, possibly acting as a molecular ratchet. Finally, we show that mutations in two residues from the Zn^2+^ binding domain, F420A and D422A, which are conserved across all RecQ family members (D422 is either D or E), result in WT DNA binding activity yet significantly reduced helicase activity and whilst F420A shows a ∼2-fold reduction in DNA stimulated ATPase activity, the D422A mutant is broadly similar to the wild-type construct. It is not clear from the structures precisely what these two residues could be doing, although they are both located at the interface between the N and C-terminal helicase lobes, possibly involved in positioning of the AR loop and the close proximity of F420A to helicase motif III may explain its reduction in ATPase activity due to the fact that motifs II and III are required for the proper stimulation of ATPase activity by DNA. In contrast to the changes in helicase activity none of the RECQL5 variants appeared to have a marked effect on the strand annealing activity when measured using a splayed duplex substrate (Supplementary Figure S8A), although it remains a possibility that variants with reduced DNA binding affinity may show a concomitant reduction in strand annealing if measured at concentrations at or below the DNA binding *K*_d_. We have also measured the strand annealing activity of RECQL5 using a double stranded DNA substrate and find significantly less annealing activity when measured using the same enzyme concentration (100 nM) (Supplementary Figure S8B), presumably reflecting the high affinity of RECQL5 for splayed duplex DNA.

### Comparisons with other RecQ family structures

**Figure 7.**
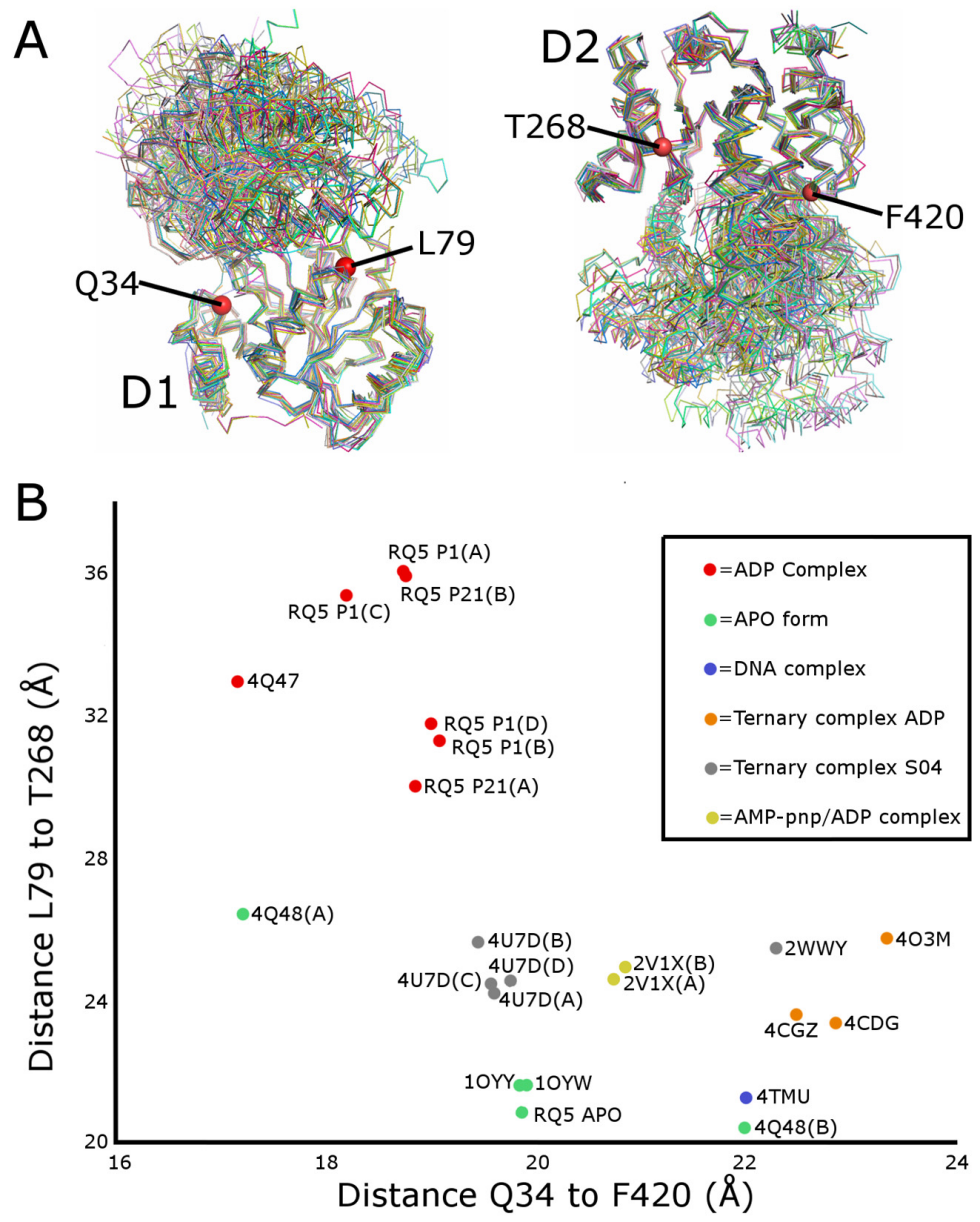
Quantitative comparison of the various RecQ family structures. (**A**) Multiple structural superposition of the current RecQ family structures aligned on the basis of the D1 domain (shown on the left) or the D2 domain (shown on the right) reveal structurally invariant positions from which to measure the relative domain positions. (**B**) 2D Plot of the D1–D2 conformation of the various RecQ family members as a function of the length of vectors between the invariant points. Individual chains of the same entry are shown separately where significant differences exist in their orientations.

The relative wealth of structural data on the RecQ family generated in recent years has offered valuable insights into the complex sequence of binding events and conformational changes that are required to couple the energy of ATP hydrolysis to DNA unwinding. However the analysis of these various structures, and the conformational space they represent is becoming more and more complex, with simple pairwise comparisons having difficulties distinguishing local structural differences from global domain movements or shifts. For this reason we have decided to implement a relative coordinate system to uncouple the local differences and analyse the relative positioning of the D1 and D2 domains in a qualitative manner across the entire RecQ family. To construct this analysis the structures were first aligned on the basis of the D1 and D2 domain alone and two invariant points on each domain chosen to represent the domain in 3D space (Figure 7A). For the D1 domain, the Cα of Leu 79 (part of motif Ia), and the Cα of Q34 (part of motif 0) were chosen and for the D2 domain the Cα of Thr 268 (part of motif IV) and Phe 420 (invariant part of the Zn^2+^ domain) were used. Distances between two pairs of these invariant points were calculated and plotted on a 2D coordinate system in Figure 7B.

From the plot it is clear that the various RecQ family structures fall into distinct groupings, with quite strong correlations between conformational states, and nucleotide and DNA binding. The ‘open’ ADP bound states of RECQL5 can be seen to group together in the upper left section of the chart along with ADP bound *D. radiodurans* RecQ (41) (PDBid 4Q47). Similarly, the ‘closed’ APO structures cluster at the bottom of the plot with RECQL5 being similar to the APO form of *E. coli* RecQ (42), and chain B of APO *D. radiodurans* RecQ (41) (chain A is in a rather more open state). The BLM and RECQ1 structures are somewhat intermediate, with the BLM structures being ternary complexes with ADP and either DNA or a Nanobody (32,33), and are further constrained by the influence of the HRDC which binds in a cleft in between the D1 and D2 domains in a nucleotide dependent manner (32). The RECQ1 DNA complex structures both contain a sulfate ion bound in the nucleotide binding site (46) in a way that mimics ADP/ATP and residues in motif I are in the same conformation as the nucleotide bound complexes, whilst the RECQ1 ADP complex (PDB id: 2V1X) was crystallized in the presence of ATP-γS which was subsequently hydrolyzed to ADP (44).

### Mechanism for RECQL5 helicase activity

A general ‘inchworm’ type catalytic mechanism has been suggested for RecQ helicases based on structural and mechanistic studies and similarities with other helicase families. However, the precise ordering and nature of the structural transitions accompanied by ATP binding hydrolysis and release both in the presence and absence of DNA has yet to be elucidated and may not be fully conserved across RecQ family members. For example, kinetic studies show that for *E. coli* RecQ ATP hydrolysis is the rate limiting step (47), while in human BLM a conformational change required for the release of ADP is the rate limiting step (48).

**Figure 8.**
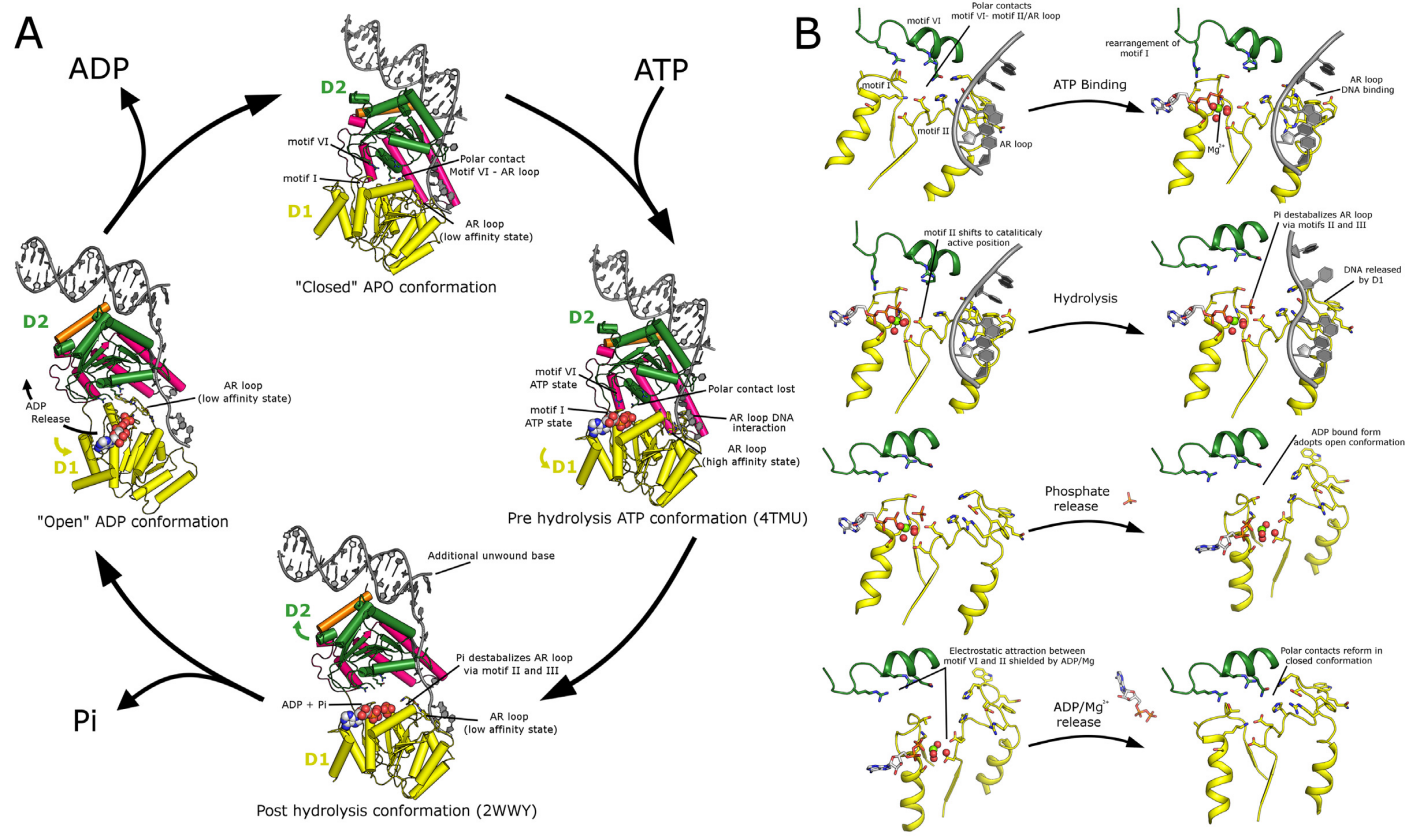
RECQL5 helicase mechanism. Helicase activity is believed to be derived by switching between distinct conformational states driven successively by ATP binding, hydrolysis, Pi release and ADP release. The AR loop in the D1 domain is capable of both modulating the affinity of the D1 domain to DNA and stimulating ATPase activity. The AR loop conformation is sensitive to polar contacts to motif IV on the D2 domain and the nucleotide/Mg^2+^ binding status. Panel (**A**) shows an overview of the entire cycle with each of the four conformational states being linked to a representative PDB entry. Panel (**B**) shows a detailed view of the changes and transitions of the conserved catalytic helicase motifs in response to ATP binding, hydrolysis and release.

We have constructed a possible model for the RECQL5 catalytic mechanism based on the transition between possible open, intermediate and closed forms which is driven by ATP binding, hydrolysis and release (Figure 8A and Bsupplemental movie 1). In our model, the conformational changes are linked via conserved helicase motifs to distinct states of the AR loop which is able to confer high affinity binding to the D1 domain and stimulate hydrolysis.

The APO form is believed to bind to DNA primarily via D2, with motif VI forming polar contacts to the AR loop stabilizing the low affinity state. ATP binding triggers a rearrangement of motif I (helix to coil transition) that would create a steric clash with motif VI forcing the two domains apart and breaking the polar contacts to the AR loop, triggering its rearrangement to the high affinity state. The rearrangement of the AR loop was found in the structure of *C. sakazakii* RecQ DNA complex (45) to cause a slight shifting of motif II (containing the catalytic Glutamate residue) closer to the position of the gamma phosphate, possibly stimulating ATP hydrolysis. In the post hydrolysis conformation a further opening of the D1 and D2 domains results in a shifting of D2 a single nucleotide step along the DNA, and the hydrolyzed phosphate is assumed to again influence the AR loop by a similar but opposite mechanism to the activation step above, causing a transition back to the low affinity conformation. We assume that phosphate is released before ADP and this may cause another transition to the open ADP bound conformation. Finally following ADP release the relatively long range charge interactions between the acidic residues on motif II and the basic residues on motif IV (which are no longer shielded by the nucleotide or magnesium ions), would bring the two domains close together to form the closed conformation again.

## CONCLUSIONS

In this study, we have determined the structures of ‘open’ and ‘closed’ forms of RECQL5 (with and without ADP/Mg^2+^ respectively). Comparisons with other RecQ family structures show the ‘closed’ APO form to be more typical of the conformation found in other structures, while solution studies show that the ‘open’ ADP form predominates in solution. We have characterized RECQL5 binding to various DNA substrates and show a fairly strong preference for binding splayed arm or looped duplex DNA structures, both of which are implicated in transcription. We show that this specificity is in part conferred by a single negatively charged helix immediately following from the Zn^2+^ domain, which is absolutely required for helicase activity in our assays, and occupies an approximately equivalent position to wedge features in other RecQ helicase and may provide the same function.

We have performed site directed mutagenesis of various residues identified in our structures to participate in conformational changes, and have measured the DNA binding, ATPase and helicase activities of these variants. We show that two residues in the AR loop W165 and H167, are important for DNA binding and essential for helicase activity although surprisingly both have DNA stimulated ATPase activity similar to WT. Instead mutations to residues in motif VI which are also required for helicase activity reduce the DNA stimulated ATPase activity most significantly. We also show for the first time that two specific residues in the Zn^2+^ domain (F420 and D422) found on the interface with the AR loop, which are strictly conserved across the RecQ family (D422 is either D or E),are required for efficient helicase activity, although their precise role is not yet clear.

Finally have used the open and closed forms of RECQL5 together with a quantitative comparison of all current RecQ family structures to construct a mechanistic model for RECQL5 helicase activity. In this model, we have attempted wherever possible to propose how the various different nucleotide states may influence the conformational status or DNA binding affinity of the various domains. The fact that RECQL5 is a relatively simplified system, lacking various accessory domains found in other SF2 helicases mean that aspects of this model may apply to other systems.

## ACCESSION NUMBERS

wwPDB entry 5LB3, wwPDB entry 5LB5, wwPDB entry 5LB8.

## Supplementary Material

Supplementary Data
