# Supplementary material for: Insights into the RecQ helicase mechanism revealed by the structure of the helicase domain of human RECQL5
Source: Nucleic Acids Res. 2017 Jan 18;45(7):4231–43. doi: 10.1093/nar/gkw1362 (PMC5397160; doi:10.1093/nar/gkw1362)
Supplement: Supplementary Data [file gkw1362_Supplementary_Data.zip › nar-02748-h-2016-File011.docx]

**Supplementary Movie S1**

The movie shows a single cycle of ATP binding, hydrolysis, phosphate and ADP release. At each stage of the cycle key residues in motifs I, II and the AR loop (shown in stick format) link the nucleotide status to distinct DNA binding conformations allowing a ratcheting mechanism and unwinding of a single base per ATP hydrolyzed (related to Figure 8).
