# Supplementary material for: Insights into the RecQ helicase mechanism revealed by the structure of the helicase domain of human RECQL5
Source: Nucleic Acids Res. 2017 Jan 18;45(7):4231–43. doi: 10.1093/nar/gkw1362 (PMC5397160; doi:10.1093/nar/gkw1362)
Supplement: Supplementary Data [file gkw1362_Supplementary_Data.zip › nar-02748-h-2016-File010.pdf]

Supplementary information

Insights into the RecQ helicase mechanism revealed by the structure of the helicase domain of human RECQL5

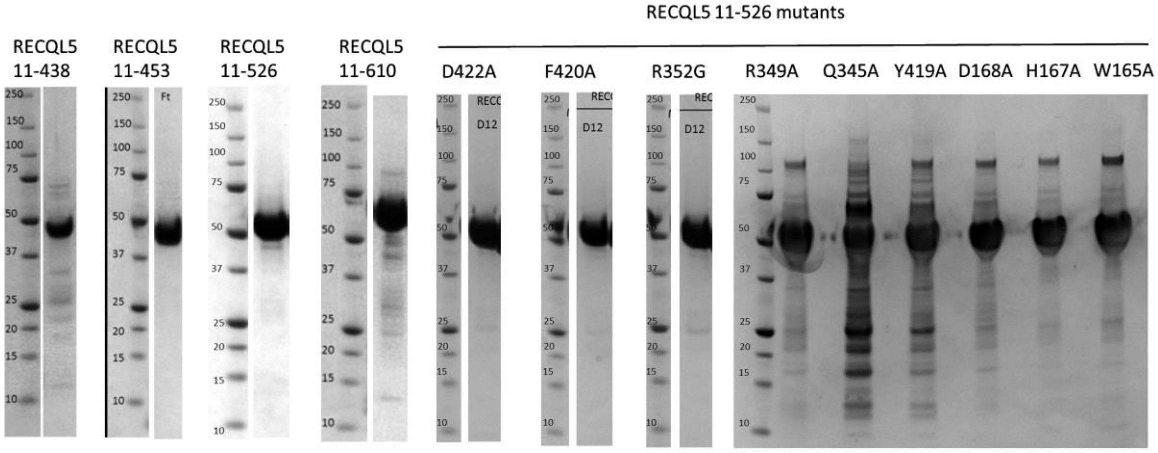

Figure S1 Coomassie stained SDS page gels of the RECQL5 constructs and variants used in this study.

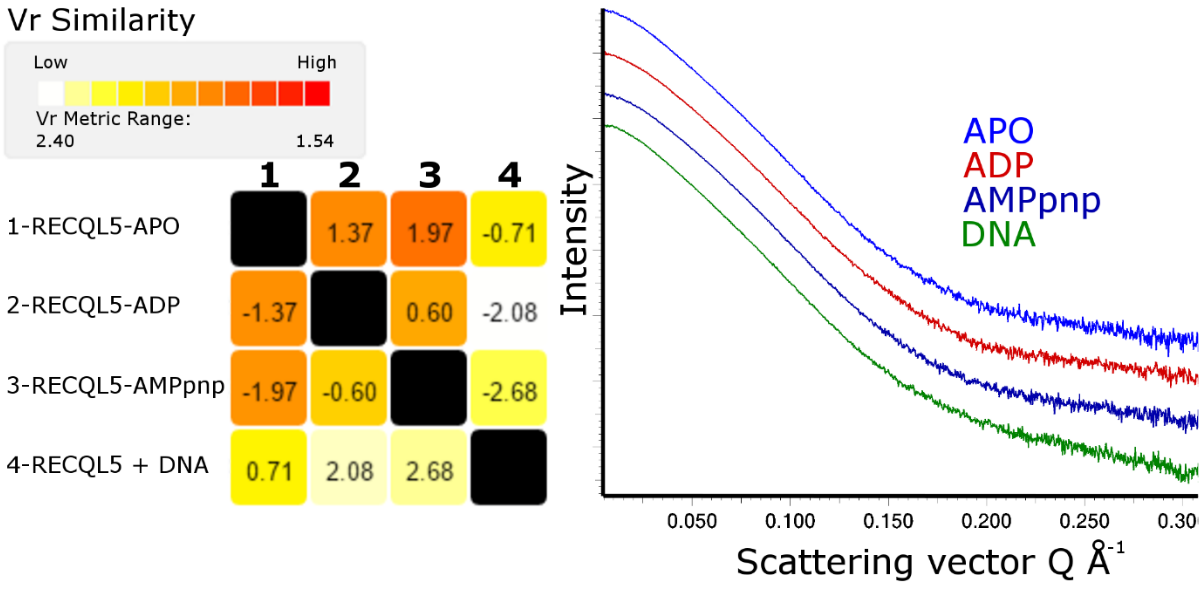

Figure S2 – Comparison of the SAXS scattering curves for RECQL5A 11-453 in the presence and absence of various nucleotides and DNA. The left hand panel shows a quantitative comparison using the programme Vr, with the raw scattering curves shown on the right.

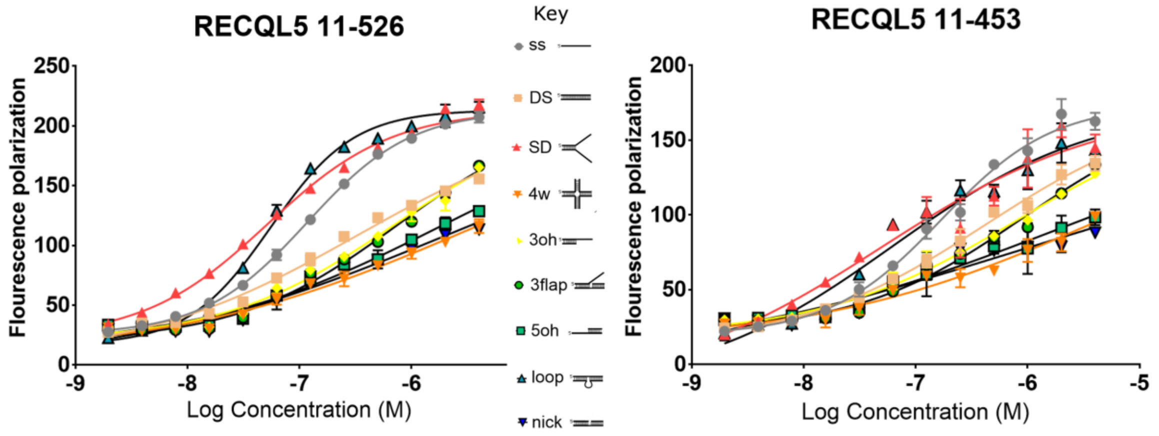

Construct	SS	DS	SD	4W	3oh	3Flap	5oh	loop	Nick
11-609	66 ±6	110 ±5	30 ±4	2320 ±328	534 ±60	550 ±47	2320 ±610	38 ±4	6511 ±1760
11-526	117 ±8	330 ±57	51 ±5	2790 ±535	680 ±63	730 ±59	1650 ±230	55 ±3	1780 ±472
11-453	169 ±22	420 ±495	50 ±24	3800 ±1290	870 ±190	900 ±171	1800 ±1140	61 ±21	900 ±5740
11-438	305 ±18	833 ±30	164 ±35	1040 ±50	590 ±28	620 ±61	940 ±46	267 ±32	1350 ±137

<100 nM	<200 nM	<500 nM	>500 nM

Figure S3 – DNA binding affinity of RECQL5A 11-526 (shown on the left) and 11-453 (shown on the right) to a variety of DNA substrates measured by fluorescence polarization. The bottom table shows a summary of the apparent dissociation constant for all constructs and substrates.

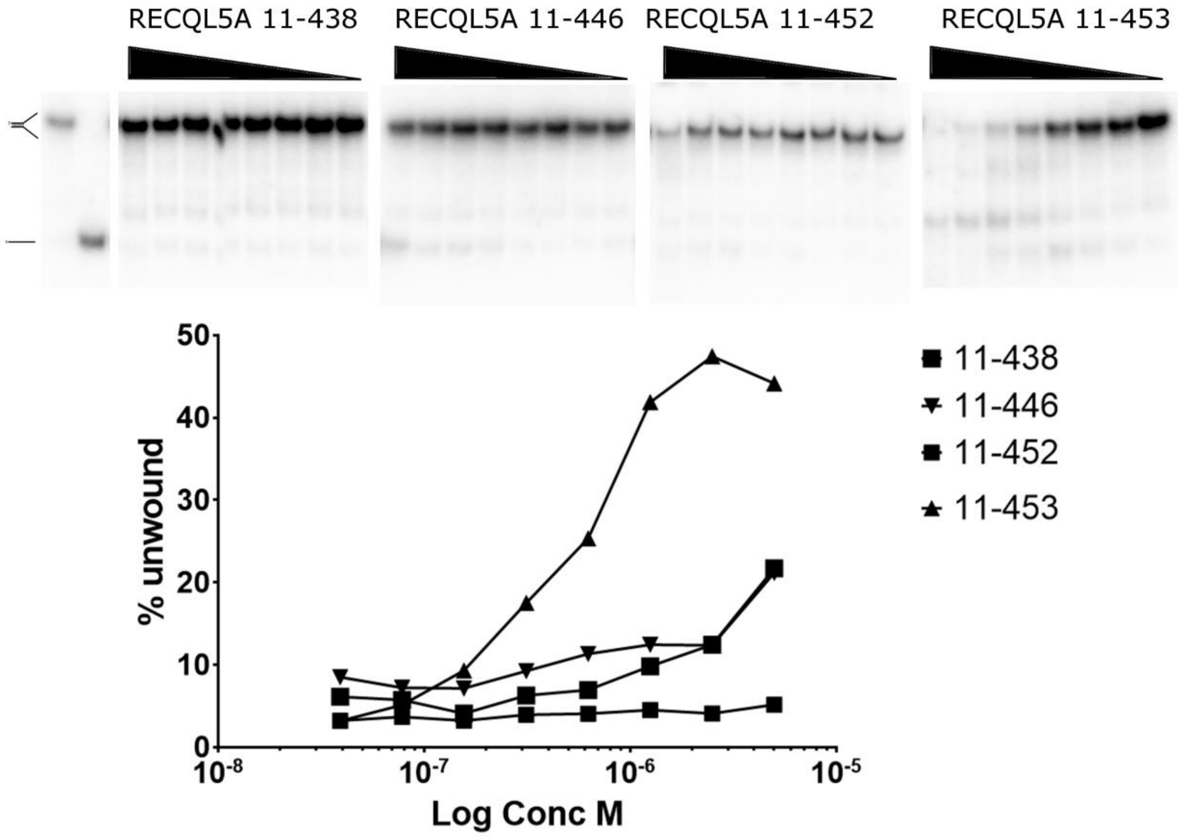

Figure S4 Helicase assays on RECQL5 wedge helix mutations, the first and second lanes are without enzyme and boiled samples respectively. The lower panel shows a graph of the quantification of the above data.

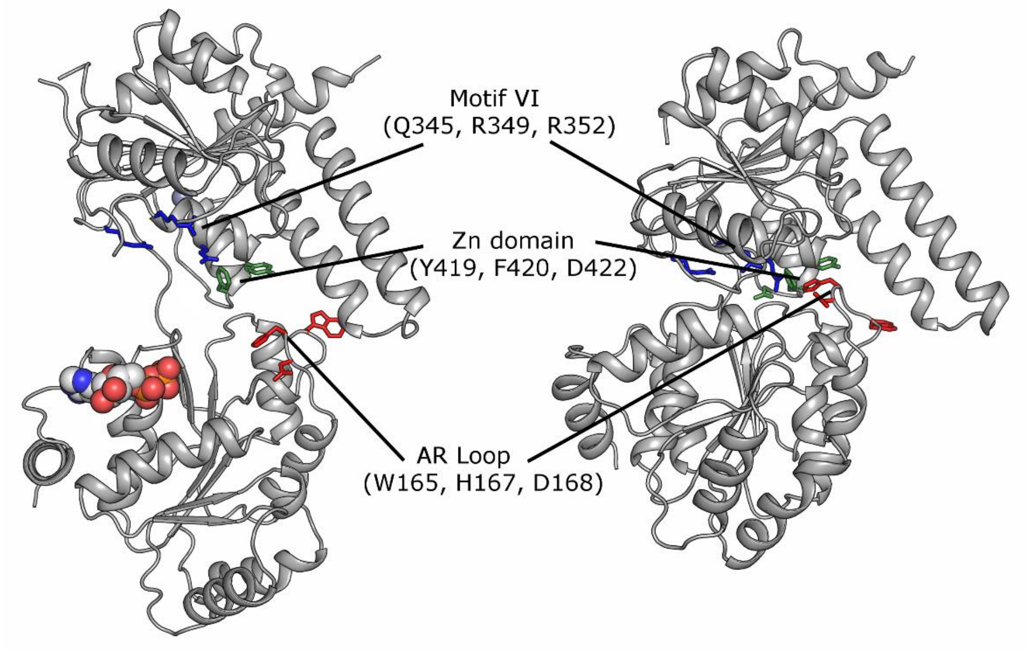

Figure S5 – Location of the residues chosen for mutagenesis residues in the open (left) and closed (right) forms of RECQL5.

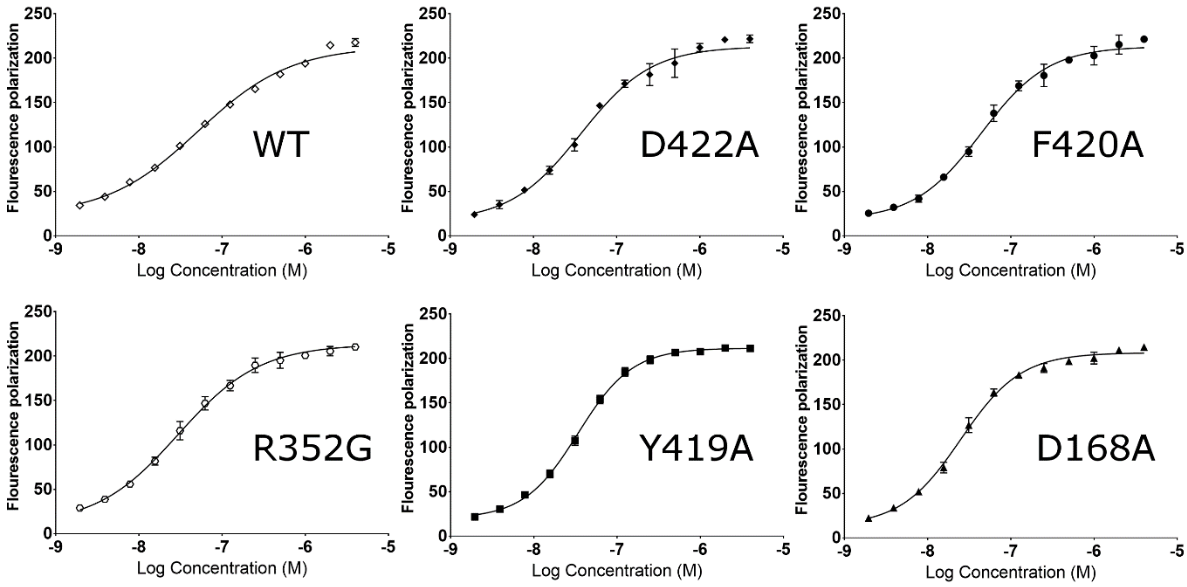

Figure S6 - Fluorescence polarization assays of RECQL5 variants that do not affect DNA binding as measured against the splayed duplex DNA substrate.

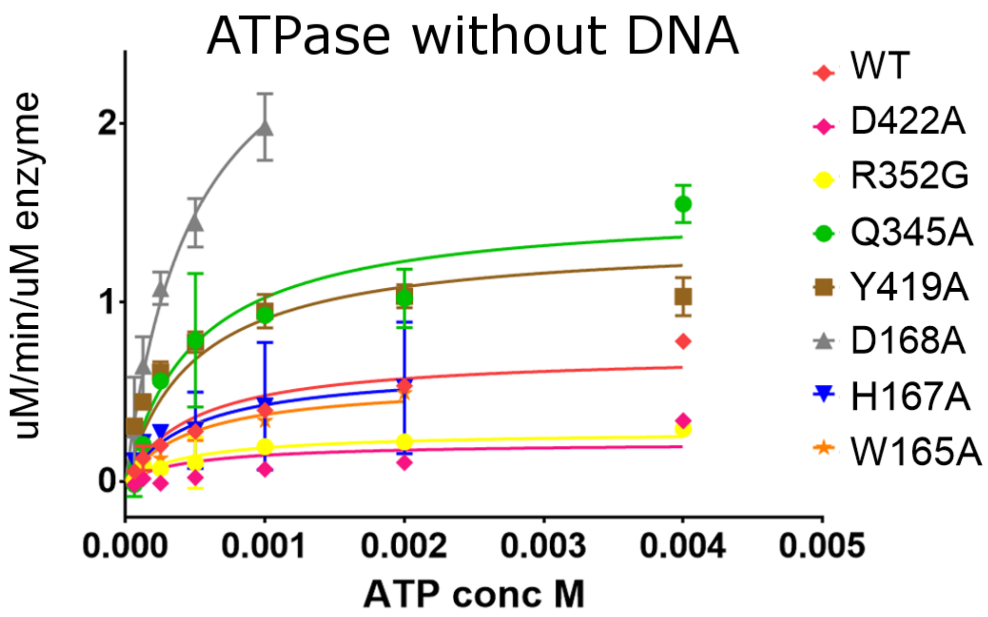

Figure S7 – ATPase activities of RECQL5 variants assayed without the stimulation of single stranded DNA.

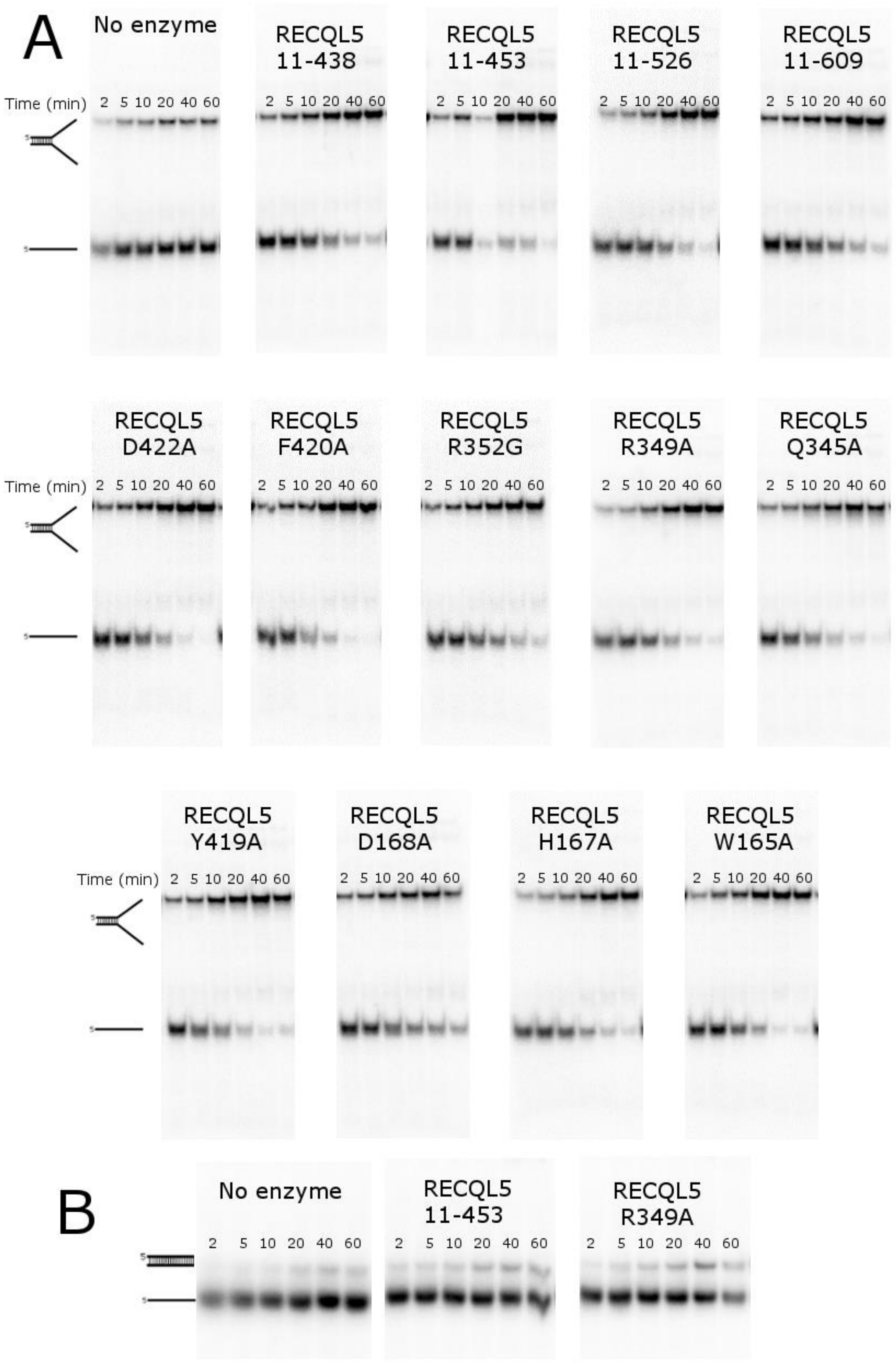

Figure S8 (**A**) DNA strand annealing activities of the RECQL5 variants, the upper left panel shows the extent of annealing over the time course of the experiment in the absence of enzyme. None of the variants showed significant differences in strand annealing. (**B**) DNA strand annealing using probes designed to give a double stranded DNA product.

Table S1A Sequences of oligonucleotides used in the fluorescence polarisation DNA binding assay

Oligonucleotide name	Sequence
RQ-1	*5'ATCGATAGTCGGATCCTCTAGACAGCTCCATGTAGCAAGGCACTGGTAGAATTCG GCAGCGTC (63 bases)
RQ-2	5'GACGCTGCCGAATTCTACCAGTGCCTTGCTACATGGAGCTGTCTAGAGGATCCGAC TATCGAT (63 bases)
RQ-3	5'GACGCTGCCGAATTCTACCAGTGCCTTGCTAGGACATCTTTGCCCACCTGCAGGTTC ACCC (61 bases)
RQ-4	5'GGAGCTGTCTAGAGGATCCGACTATCGA (28 bases)
PQ-5	5'TGGGTGAACCTGCAGGTGGGCAAAGATG (28 bases)
PQ-6	5'GGGTGAACCTGCAGGTGGGCAAAGATGTCCATTAGTGGATCCTTAGCACCGTTGT AAGACG (61 bases)
PQ-7	5'CGTCTTACAACGGTGCTAAGGATCCACTAATCATGGAGCTGTCTAGAGGA TCCGACTATCGAT (63 bases)
PQ-8	5'GACGCTGCCGAATTCTACCAGTGCCTTGCTATTTTTTTTTTTTTCATGGAGCTGTCTAG AGGATCCGACTATCGAT (75 bases)
PQ-9	5'GACGCTGCCGAATTCTACCAGTGCCTTG (28 bases)

* Oligonucleotide RQ-1 was obtained with a Fluorescein isothiocyanate label on the 5' end

Table S1B Mixing scheme for DNA substrates

Substrate name	Structure	Oligonucleotides
Single stranded (SS)	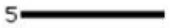	RQ-1
Double stranded (DS)	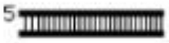	RQ-1 + RQ-2
3’ Overhang (3oh)	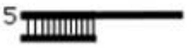	RQ-1 + RQ-4
Splayed duplex (SD)	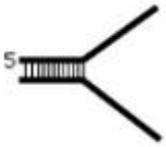	RQ-1 + RQ-3
3’ Flap (3Flap)	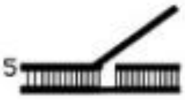	RQ-1 + RQ-3 + RQ-4
4 Way junction (4W)	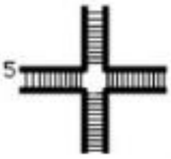	RQ-1 + RQ-3 + RQ-6 + RQ-7
Looped duplex (loop)	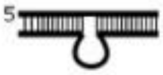	RQ-1 + RQ-8
5’ overhang (5oh)	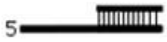	RQ-1 + RQ-9

In all cases the fluorophore was attached to the 5’ end of oligo RQ-1 in the position marked by the 5 on the structure diagrams.
